# Preparation and Characterization of Chitosan/β-Glycerophosphate Thermal-Sensitive Hydrogel Reinforced by Graphene Oxide

**DOI:** 10.3389/fchem.2018.00565

**Published:** 2018-11-22

**Authors:** Han Qin, Jian Wang, Tong Wang, Xiaomeng Gao, Qianbing Wan, Xibo Pei

**Affiliations:** State Key Laboratory of Oral Diseases, Department of Prosthodontics, National Clinical Research Center for Oral Diseases, West China Hospital of Stomatology, Sichuan University, Chengdu, China

**Keywords:** thermal-sensitive, hydrogel, chitosan, β-glycerophosphate, graphene oxide

## Abstract

Thermal-sensitive hydrogel based on chitosan (CS) and β-glycerophosphate (GP) has shown good biocompatibility and biodegradability. But the application of such hydrogel is limited due to its poor mechanical property. Recently, graphene oxide(GO) is widely used as a reinforcement agent to prepare nanocomposites with different polymers for improving the properties of the materials. In this study, CS/GP-based hydrogels with different weight ratio of GO/CS (0.5, 1, 2%) were fabricated. The gelation time of the hydrogels at body temperature was evaluated by tube inverting method. The gelation process during heating was monitored by rheological measurement. The morphology, porosities, chemical structure, swelling properties of the lyophilized hydrogels were investigated by scanning electron microscopy, liquid displacement method, Fourier transform infrared spectroscopy and gravimetric method. Mechanical property of the hydrogels was analyzed by rheological measurement and unconfined compression test. MC3T3-E1 mouse pre-osteoblast cell line was used to assess the biological properties of the hydrogels. The results obtained from those assessments revealed that the addition of GO into CS/GP improved the properties of the prepared hydrogels without changing the high porous and interconnected microstructure and swelling ability of the hydrogels. The gelation time at body temperature was significantly reduced by nearly 20% with the addition of small amount of GO (0.5% weight ratio of CS). The mechanical properties of the hydrogels containing GO were improved significantly over that of CS/GP. The storage (G′)/loss (G″) moduli of the hydrogels with GO were 1.12 to 1.69 times that of CS/GP at the gelling temperature. The Young's modulus of 0.5%GO/CS/GP hydrogel is 1.76 times that of CS/GP. Moreover, the 0.5%GO/CS/GP hydrogel revealed remarkable biological affinity such as cellular attachment, viability and proliferation. All of these results suggest that 0.5%GO/CS/GP hydrogel has great potential for practical application in biomedical field.

## Introduction

Hydrogels have some unique physical properties, which make them widely used in biomedical fields such as tissue engineering, wound dressing and drug delivery systems (Bhattarai et al., 2010). A good candidate to form hydrogel is chitosan (CS), a copolymer of β[1,4]-linked 2-acetamido-2-deoxy-D-glucopyranose and 2-amino-2-deoxy-D-glucopyranosea. This biopolymer with polycationic structure is generally obtained by alkaline deacetylation from chitin which is the second most abundant polysaccharide in nature after celluse (Rinaudo, 2006). Because of its non-toxicity, biocompatibility, biodegradability, antibacterial ability, and low immunogenicity, chitosan has attracted large interest in biological application such as tissue engineering, drug delivery and wound healing (Riva et al., 2011; Zhao et al., 2015, 2018; Dong et al., 2016). These admirable characteristics of chitosan is ascribed to its unique chemical structure. For example, the polysaccharide unit of chitosan resembles the structure of glycosaminoglycans (GAGs), which is the major component of the extracellular matrix (ECM) of bone and cartilage (Khor and Lim, 2003). The cationic character of chitosan enables it to form complexes with anionic molecules, including proteoglycans, GAGs, growth factors, receptors and adhesion proteins that help to regulate cellular activity (Lehr et al., 1992; Dutta et al., 2011). In addition, chitosan can create networks with highly porous and interconnected pore structure which is helpful for cell adhesion, proliferation, and differentiation as it enhances the diffusion of nutrients and provide room for neovascularization (Dutta et al., 2011). Hence, chitosan can be used to form hydrogels as scaffolds or substrate for tissue engineering.

Generally, chitosan hydrogels can be prepared by either covalently or ionically crosslinking methods with the addition of different crosslinkers. Covalently cross-linked hydrogels possess a stable and permanent network structure as irreversible chemical bonding are formed. However, covalent cross-linkers such as glutaraldehyde are usually toxic to organism. And the chemical crosslinking makes hydrogels hard to degrade. On the other hand, ionically cross-linked hydrogels are formed by reversible links and generally considered biocompatible, exhibiting a higher swelling sensitivity to pH changes compared with covalently cross-linked chitosan hydrogels (Berger et al., 2004). Because chitosan is a polycationic polymer, one of the anionic molecules—β-glycerophosphate (GP) can be used as an ionic crosslinking agent. It is an organic compound naturally found in the body and has been used as an osteogenic supplement for culturing human bone marrow stem cells (Zhou et al., 2015). Chitosan/β-Glycerophosphate (CS/GP) is an *in situ* thermal-sensitive gelling system, which maintains fluidity at low temperature (room temperature or below) and becomes gel at higher temperature (body temperature) (Berger et al., 2004; Zhou et al., 2015). With this characteristics, CS/GP hydrogel has attracted great attention as it does not require an surgical operation when implanted (Zheng et al., 2010). However, the usage of the material is usually limited due to its poor mechanical properties. Hence, many studies aimed at improving its mechanical functionality by blending CS/GP system with other nanomaterials, such as nano-attapulgite (Wang and Chen, 2016), nano-hydroxyapatite (Huang et al., 2011; Chen et al., 2016), nanosilver (Tsai et al., 2011), carbon nanotubes(CNTs) (Gholizadeh et al., 2017) and etc.

In addition to those nanofillers, a recently attractive material has yet not been studied as a reinforcing agent in CS/GP system—graphene. Graphene is expected for application in various fields because of its exceptional physico-chemical properties (Zhu et al., 2010). It is considered as a promising nanofiller as it possesses high aspect ratio which is a very useful parameter for improving the properties of materials, such as thermal, electrical and mechanical properties (Stankovich et al., 2006; Cote et al., 2009; Salavagione et al., 2009; Vickery et al., 2009). Compared with its allotrope CNTs, graphene offers enhanced mechanical properties to the composite, due to the planar structure and the aspect ratio, which allows better stress transfer within the host medium during the loading process (Mittal et al., 2015). Moreover, graphene-based materials promote stem cell attachment and growth, and enhance osteogenic differentiation, indicating its application as an alternative material for bone regeneration research (Dubey et al., 2015). A widely used form of graphene is graphene oxide (GO), which is amphiphilic due to the multiple oxygen-containing groups such as hydroxyls, epoxides, and carboxyls on its surface (Li et al., 2008; Marcano et al., 2010). These functional groups (oxygen-containing) allow GO to disperse at an individual sheet and highly negatively charged in aqueous solution, which makes it possible to form special interaction between GO and protonated polymers such as CS. Meanwhile, these functional groups (oxygen-containing) as well as the large aromatic (π-configuration) interface endow GO with the ability to interact with proteins, peptides, or DNA via chemical bonding or physical adsorption (Shin et al., 2016). Adding GO into CS to form films or scaffolds have been reported with improved properties, including mechanical strength in both wet and dry state, storage modulus, and thermal stability (Fan et al., 2010; Yang et al., 2010; Depan et al., 2011; Han et al., 2011; Dinescu et al., 2014). Because of the advantages of graphene and its derivatives and previous studies of CS and GO mentioned above, we considered adding graphene oxide into CS/GP system to improve the properties of CS/GP hydrogel.

In the present work, GO/CS/GP hydrogels with different GO/CS weight ratios were prepared as the amount of GO in nanocomposites plays an important role to the properties of materials. Meanwhile, the physical, chemical and biological properties of each hydrogel were evaluated. We expect to develop a composite hydrogel of GO/CS/GP with suitable mechanical properties while maintaining biological functionality for biomedical applications.

## Materials and methods

### Materials

β-Glycerophosphate disodium salt was obtained from Sigma-Aldrich Co.(US). Medium molecular weight chitosan(molecular weight≈21,0000 Da, deacetylation degree≥95%) was purchased from Tokyo Chemical Industry Co., Ltd. Acetic acid (chemical reagents of analytical grade) was provided by Baoxin Bio-Technology Co., Ltd. (Chengdu, China). Single-layered graphene oxide, purity>99%, diameter of 0.5–5 μm, thickness of 0.8–1.2 nm was purchased from Nanjing/Jiangsu XFNANO Materials Tech Co., Ltd. (Nanjing, China).

### Preparation of the hydrogels

To prepare 10 mL GO/CS/GP hydrogel, the following steps were implemented. Firstly, 200 mg CS powder was dissolved in 6 mL acetic acid (0.75%v/v) and vigorously stirred for 1 h; prescribed amount (1, 2 or 4 mg) of GO sheets were dispersed in 2 mL distilled water and treated by ultrasonication for 1 h; 600 mg GP was dissolved in 2 mL distilled water. Secondly, GO dispersion was added into the CS solution and stirred for 1 h until complete mixture. Finally, the GP solution was added dropwise to the GO/CS mixed solution under constant stirring. As the control group, the CS/GP hydrogel was prepared following the same steps above except that the GO dispersion was replaced by 2 mL distilled water. According to the weight ratio of GO and CS, the samples were grouped as CS/GP, 0.5%GO/CS/GP, 1%GO/CS/GP, and 2%GO/CS/GP. The total concentrations of the components in each hydrogel groups were shown in Table 1.

**Table 1:** Total concentrations of component in each hydrogel group.

**Group**	**Chitosan wt %**	**GO wt %**	**GO/CS weight ratio %**	**GP wt %**
CS/GP	2	0	0	6
0.5%GO/CS/GP	2	0.01	0.5	6
1%GO/CS/GP	2	0.02	1	6
2%GO/CS/GP	2	0.04	2	6

### Gelation time determination

Appropriate gelation time of the hydrogel is of much importance for the clinical or biomedical application. The test tube inverting method was used to measure the gelation time at constant temperature of 37°C in a water bath (Zhao et al., 2009; Mirahmadi et al., 2013). One milliliter of each sample (*n* = 5) was added into test tubes at room temperature, then incubated in the water bath. The fluidity of the samples was observed every 30 s by tilting the tube. The time at which flow stopped was taken as the gelation time and the values were recorded.

### Rheological measurement

To analyze the rheological properties of the hydrogels in the gelling process, rheology tests was performed with a rheometer (HAKKE Viscotester IQ Air 260-100, Thermofish, US), which required about 2 mL of the solution per sample. Samples of all groups were inserted into the rheometer. Oscillatory measurements were performed at 1 Hz, while the temperature was elevated at the rate of 2°C/min from 10 to 60°C. During the gelling process, the dynamic rheological properties such as the dynamic elastic (storage) modulus (G′) and the viscous (loss) modulus (G″) were measured by oscillatory shear measurement. The gelation temperature was determined as the crossover point of the elastic (G′) and viscous (loss) moduli (G″) (δ = 45°).

### Scanning electron microscopy (SEM)

After gelation, all samples were frozen in a refrigerator at −20°C for 2 h and then lyophilized in a freeze drier at −80°C for 24 h. Afterwards, the samples were sputter coated with gold and scanning electron microscopy (SEM) (Inspect F50, FEI, Hillsboro, OR, US) was used to evaluate the surface morphology, pore morphology, and pore size distribution of the samples. The average pore size of each sample were obtained using the software Digimizer, 20 pores were considered.

### Porosity determination

The porosities of the hydrogels were measured using liquid displacement method. Briefly, initial weight and volume of the lyophilized hydrogels (*n* = 5) were measured and recorded (*W* and *V*, respectively). Then, the gels were immersed in dehydrated alcohol for 24 h until they were fully saturated. After removal of the surface liquid, the gels were weighted again (*W'*). The porosities were calculated using the equation below (Gholizadeh et al., 2017):

$$\begin{array}{l} \text{Porosity}\left(\%\right)=\left[\left(W'-W\;\right)/\left(V\times \rho \right)\right]\;\times 100\% \end{array}$$

Where ρ is the density of alcohol.

### Fourier-transform infrared (FTIR) spectroscopy

The FTIR spectra of CS, GO, GP and all the lyophilized samples were recorded in KBr pellets with a Nicolet FT-IR 6700 spectrophotometer (Thermo Nicolet Corp., Madison, WI, US). An IR spectral range of 400–4,000 cm^−1^ was analyzed.

### *In vitro* swelling kinetic analysis

The water uptake ability of the hydrogels was measured by classical gravimetric method (Mirahmadi et al., 2013; Gholizadeh et al., 2017). Each sample (*n* = 5) of lyophilized hydrogels was weighed (*W*_d_) and immersed into the distilled water at 37°C for 12 h. At appropriate intervals (10, 20, 30 min, 1, 2, … 12 h) , samples were retrieved and blotted with filter paper to remove the surface solution and weighed again (*W*_w_). The water uptake ratio (*E*_u_) at each interval was calculated as below (Mirahmadi et al., 2013; Gholizadeh et al., 2017):

$$\begin{array}{l} E_{\text{u}}=\left[\left(\;W_{\text{w}}-W_{\text{d}}\right)/\;W_{\text{d}}\right] \end{array}$$

### Mechanical properties

The compressive mechanical property of the hydrogels was tested by a universal tensile testing machine (SANS CMT4000) at room temperature under a 9 N load cell. Dimensions of the samples were measured carefully using a digital caliper. The state of the hydrogel samples was examined with crosshead speed of 2 mm/min until reaching 20% strain. At least four specimens were tested for each group. Force and deformation data were collected by the test instrument and were converted to stress and strain values. Elastic modulus was determined from the slope of the linear portion of the stress–strain curve for all samples.

### Cell studies

The cell studies were conducted using the mouse pre-osteoblast cell line MC3T3-E1. Alpha-minimum essential medium (α-MEM, HyClone, USA) supplemented with 10% fetal bovine serum (FBS, HyClone, USA) and 1% penicillin–streptomycin was used to culture the cells.

Sterile hydrogels were prepared according to the steps mentioned above (Preparation of the hydrogels) in aseptic environment. The CS powder and GO sheets were sterilized by ultraviolet ray radiation before dissolved in acetic acid and dispersed in distilled water. The GP solutions and acetic acid were sterilized by using 0.22 μm filtration.

The hydrogel solutions were placed in 24-well plates (0.5 mL for each well) and incubated in a humidified incubator at 37°C. After gelation, all the hydrogels were washed three times with cell culture medium every 30 min and then MC3T3-E1 cells were seeded into the hydrogels (5 × 10^4^ cells/well). 1 mL complete medium was added to each well. Culture medium was changed every 2 days.

### Cell attachment

SEM was used to study the attachment and morphology of the cells on the hydrogels. The culture medium was removed from the cell-gel constructs after cells were cultured on the gels for 24 h. The cell-gel constructs were washed twice with PBS, followed by fixation with 2.5 vol.% glutaraldehyde for 2 h at 4°C. After removing the fixatives, the constructs were dried with a graded series of ethanol and sputter coated with gold. Then the samples were observed by SEM.

### Cell proliferation

CCK-8 test was used for cell proliferation assessment. After culturing for 1, 4, and 7 days, cell culture medium of the samples(*n* = 4) was removed and 350 μL fresh culture medium with 35 μL CCK-8 reagent was added to each sample. After incubated at 37°C for 2 h, 100 μL medium of each well was transferred to 96-well plate. The absorbance values were measured using a microplate reader (Bio-Rad, USA) at wavelength of 450 nm. The results obtained were expressed as optical density (OD) after blank subtraction.

### Cell viability and morphology

Fluorescent inverted microscopy was used to study the viability and morphology of MC3T3-E1 on the hydrogels using live/dead staining kit Calcein-AM/Propidium iodide (Calcein-AM/PI, Sigma, USA). Cells were cultured on the gels for 1 d and 5 d, then culture medium was removed and cell-gel constructs in each well were washed twice with PBS. Onemilliliter dye (10 μg/mL in PBS) was added into each well. After incubation for 45 min, the dye was removed and the constructs were washed once with PBS. The samples were observed with fluorescence inverted microscope (IX 71, Olympus, Japan) under blue fluorescent light (490 nm) and green fluorescent light (545 nm), respectively. Live cells were stained green and nucleus of dead cells were colored red.

### Statistics

At least three samples were tested for each experiment and all data were reported as mean ± SD. Statistical analysis was carried out using one-way analysis of variance (ANOVA) for the comparison of groups and results were set as significant for *p* < 0.05.

## Results

### Gelation time

**Figure 1:**
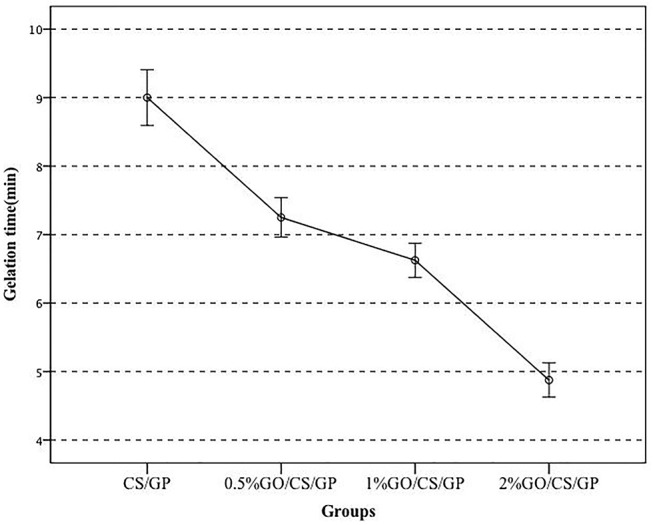
The gelation time of the hydrogels with different content of GO at 37°C.

Figure 1 showed the gelation time of the hydrogels at 37°C. All the samples were converted to gels around physiological temperature in < 10 min but the time durations were different. CS/GP became gel in 9 ± 0.41 min, while 0.5%GO/CS/GP became gel in 7.25 ± 0.29 min. The gelation time was reduced about 20% (*p* < 0.05) by adding a small amount of GO (0.5% weight ratio of CS) into the CS/GP. Gelling time appeared to display an apparent decrease with the increase of GO/CS ratio. 1%GO/CS/GP and 2%GO/CS/GP became gels in 6.63 ± 0.25 min(1%GO/CS/GP) and 4.88 ± 0.25 min(2%GO/CS/GP), respectively.

### Rheological measurement

**Figure 2:**
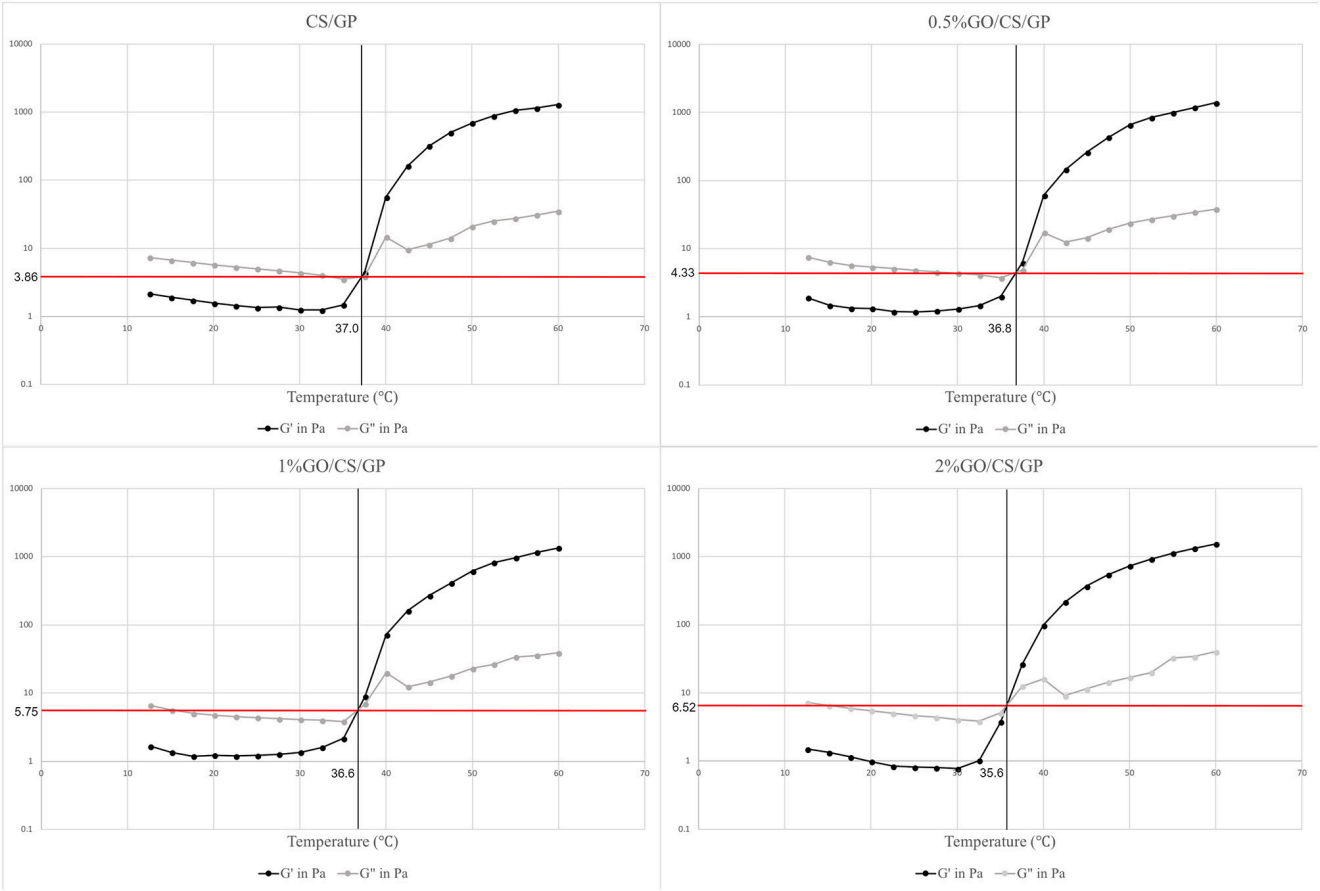
Storage modules (G′) and loss modulus (G″) of the hydrogels during gelation process. The crossover points are indicative of gel formation as G′ becomes greater than G″ in a certain temperature.

The results of rheology tests were showed in Figure 2. There were three regions in the curves according to the temperature range upon the heating process. In region 1, the samples showed a viscoelastic fluidlike behavior (G′ < G″), and both G′ and G″ moduli decreased as the temperature increased, which is the common behavior of a polymer solution. In the next region (region 2), both storage modulus (G′) and loss modulus (G″) abruptly increased during heating due to the fast formation of the three-dimensional network. However, the growing rate of G′ was much larger than that of G″ in this region, indicating that the development of the gel structure contributed to the stiffening of the system. The gelation temperature was determined at the crosspoint of storage (G′) and loss (G″) moduli ranging from 35.7 to 37.0°C. The slight difference of gelation temperature of the hydrogels may attribute to the existence of graphene oxide. At the gelling point, the storage (G′)/ loss (G″) moduli showed a positive correlation with the amount of GO. The storage (G′)/ loss (G″) moduli of CS/GP, 0.5%GO/CS/GP, 1%GO/CS/GP and 2%GO/CS/GP at the gelling point were 3.86, 4.33, 5.75, and 6.52 Pa, respectively. In the last zone (region 3), the gelation process became much slower because of the lower diffusivity resulted from the increasing viscosity during the network formation.

### Morphology observation

**Figure 3:**
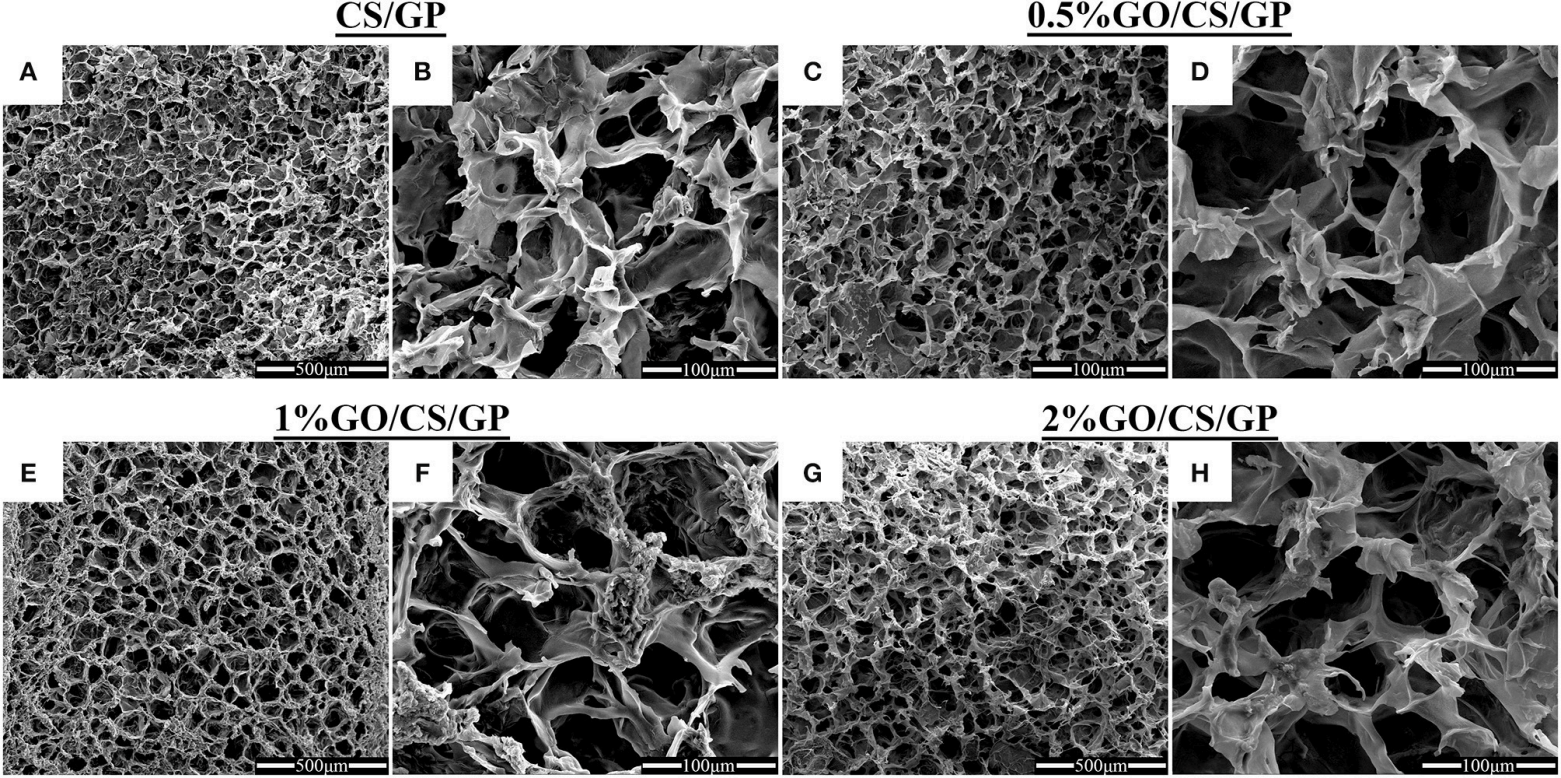
Scanning electron micrographs of CS/GP **(A,B)**, 0.5%GO/CS/GP **(C,D)**, 1%GO/CS/GP **(E,F)** and 2%GO/CS/GP **(G,H)** hydrogels illustrating the porous and interconnected microstructure.

The high porous homogeneous and interconnected structure of the lyophilized hydrogels were revealed by scanning electron microscopy (Figure 3). The microstructure of the hydrogels containing GO were similar to that of CS/GP, irrespective of the amount of GO. The average pore sizes of each hydrogel were presented in Table 2. The average pore size of CS/GP was 56.61 ± 18.82 μm, while the average pore size of 0.5%GO/CS/GP was 58.83 ± 19.85 μm. With the adding amount of GO, the average pore sizes were substantially increased to 65.26 ± 15.32 μm (1%GO/CS/GP) and 69.61 ± 19.72 μm (2%GO/CS/GP). The interconnecting pores and canals of the hydrogels were uniform and coherent, which makes fast liquid convection. Porosity measurement was conducted by liquid displacement method and the results are presented in Table 2. All the hydrogels had porosities in the range 80% to 90%. The porosity of the CS/GP was the lowest (81 ± 3%), whereas, by the addition of small amount of GO (0.5% weight ratio of CS) into the gelling system, the porosity was increased to 83 ± 2%. The porosities of 1%GO/CS/GP and 2%GO/CS/GP were 84 ± 3% and 88 ± 3%, respectively.

**Table 2:** Summary of the physical properties of lyophilized hydrogels.

**Group/properties**	**Average pore diameter (μm)**	**Porosity (%)**
CS/GP	56.61 ± 18.82	81 ± 3
0.5%GO/CS/GP	58.83 ± 19.85	83 ± 2
1%GO/CS/GP	65.26 ± 15.32	84 ± 3
2%GO/CS/GP	69.61 ± 19.72	88 ± 3

### Water uptake

**Figure 4:**
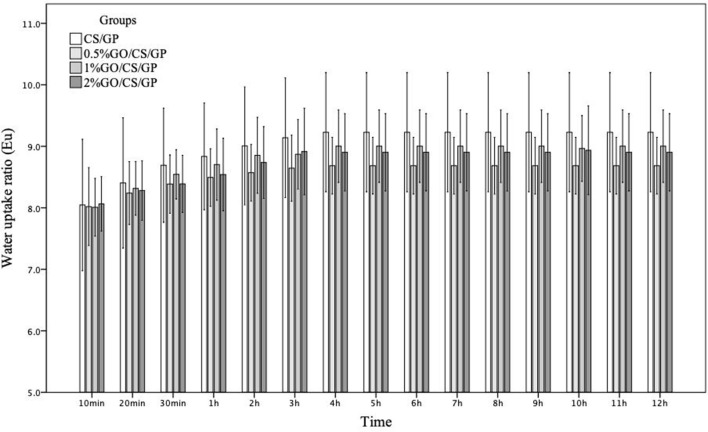
The water uptake ratios of CS/GP, 0.5%GO/CS/GP, 1%GO/CS/GP, 2%GO/CS/GP with time.

The water uptake abilities of the hydrogels were displayed in Figure 4. The swelling ratios of all the four hydrogels showed a similar trend of time-dependent increasing in the initial 4 h and then remained unchanged in the following 8 h, which indicated the hydrogels had reached their equilibrium state. In general, the water uptakes ratios of all the hydrogels were above 8 after immersion for 10 min and increased with time. At equilibrium state, the *E*_u_ of CS/GP was 9.23 ± 0.83, while *E*_u_ of 0.5%GO/CS/GP was 8.68 ± 0.33. However, there was no significant difference among *E*_u_ values of the groups.

### FTIR spectra of the hydrogels

**Figure 5:**
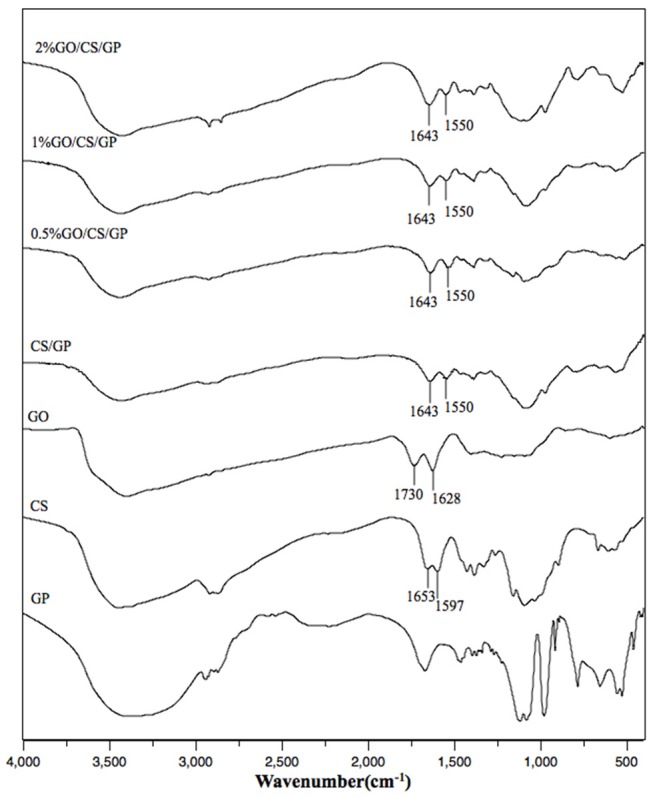
The FTIR spectrum of CS/GP, 0.5%GO/CS/GP, 1%GO/CS/GP, 2%GO/CS/GP, CS, GO, GP.

The differences in chemical structures of GP, GO, CS, CS/GP, 0.5%GO/CS/GP, 1%GO/CS/GP, and 2%GO/CS/GP determined by FTIR were shown in Figure 5. In the spectrum of GO, the peaks at 1,730 and 1,628 cm^−1^ represented C = O stretch of the carboxylic group and the C–C stretching mode of the sp^2^ carbon skeletal network, respectively. In regard to CS, two characteristic absorbance bands centered at 1,653 and 1,597 cm^−1^ corresponded to the C = O stretching vibration of –NHCO– (amide I) and the N–H bending of –NH_2_ (amide II), respectively. The spectrums of all the lyophilized hydrogels were similar, showing lower wavenumbers of amide I (1,643 cm^−1^) and amino groups (1,550 cm^−1^). This could be interpreted by the electrostatic interaction between protonated CS and negative charged GP (Zhou et al., 2008; Assaad et al., 2015). For the hydrogels containing GO, the disappearing of the peak at 1,730 cm^−1^ which related to C = O stretch could be explained by the synergistic effect of H-bonding between CS and the oxygen-containing groups in GO and electrostatic interaction between polycationic CS and the negatively charged GO (Yang et al., 2010).

### Mechanical testing

**Figure 6:**
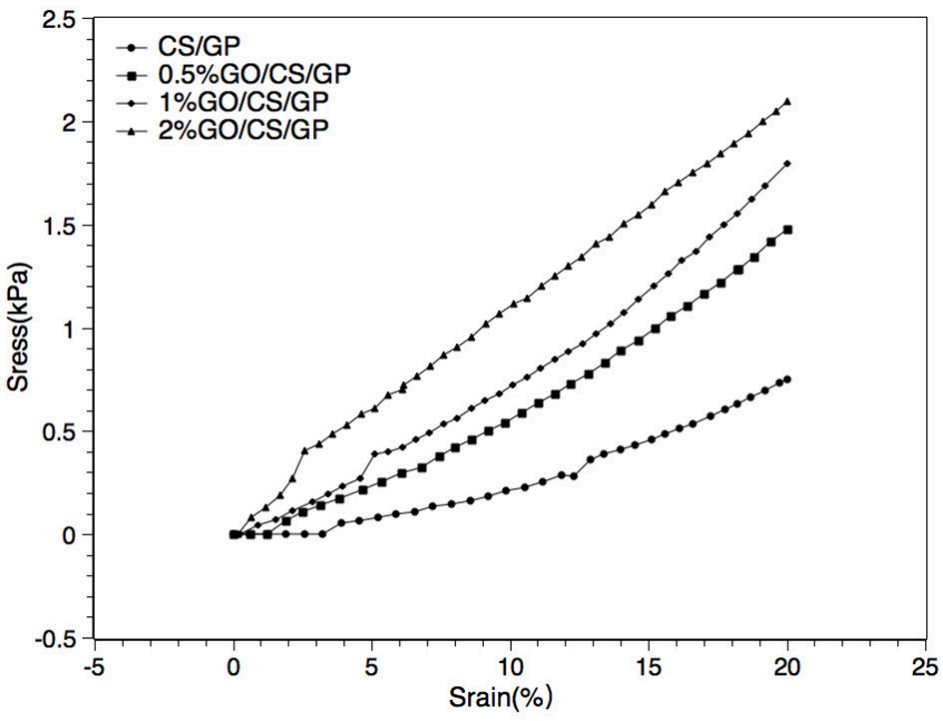
Representative strain–stress curves of CS/GP, 0.5%GO/CS/GP, 1%GO/CS/GP, 2%GO/CS/GP.

**Figure 7:**
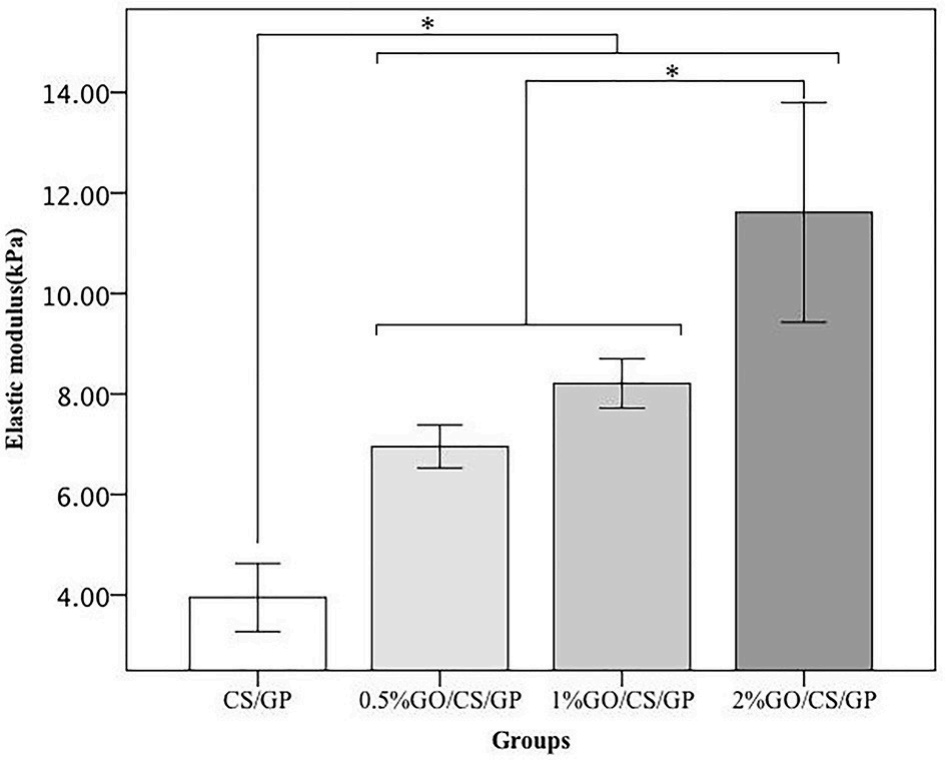
Elastic modulus obtained by unconfined compressive mechanical tests. *Statistically significant differences existing among the groups.

Results of unconfined compression test on the hydrogels were shown in Figures 6, 7. Stress-strain profiles (Figure 6) showed that all the hydrogels exhibited a linear strain-stress relationship from 0 to 20% strain. Determination of the linear modulus (Figure 7) revealed that 0.5%GO/CS/GP hydrogel was 1.76 times stiffer than CS/GP hydrogel (*p* < 0.05). 0.5%GO/CS/GP hydrogel had a modulus of 6.96 ± 0.43 kPa while CS/GP hydrogel had the modulus of 3.95 ± 0.68 kPa. The modulus of the hydrogels displayed a positive correlation with the content of GO as the modulus of 1%GO/CS/GP and 2%GO/CS/GP were 8.21 ± 0.49 and 11.61 ± 2.19 kPa, respectively.

### Cellular assay

**Figure 8:**
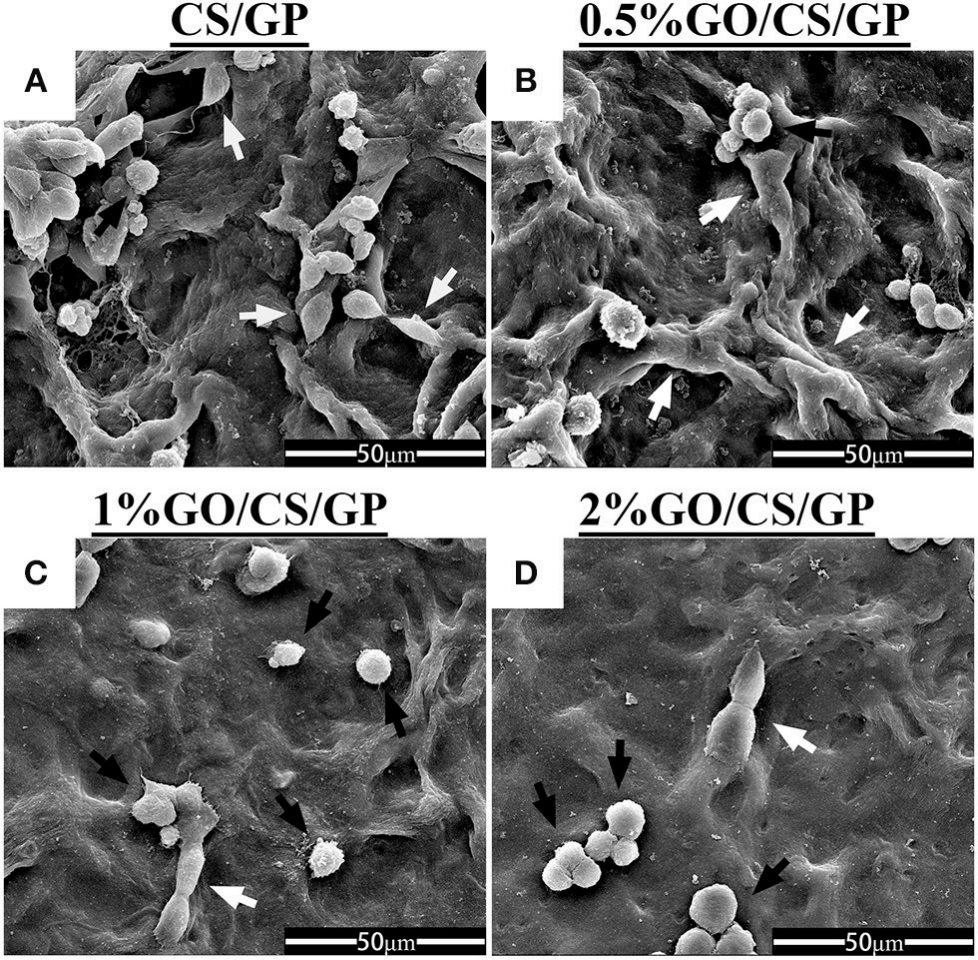
Scanning electron micrographs of cellular adhesion on CS/GP**(A)**, 0.5%GO/CS/GP**(B)**, 1%GO/CS/GP**(C)**, and 2%GO/CS/GP**(D)** after incubation for 24 h. White arrows indicate cells with spindle shape, black arrows indicate cells with spherical shape.

The SEM micrographs in Figure 8 illustrated cellular attachment on the surface of the hydrogels. The porous structure could not be observed as the dehydration treatment before SEM made the hydrogel shrink to a film. Most of the cells on the surface of the CS/GP and 0.5%GO/CS/GP hydrogels showed a spindle shape, which was the typical morphology of MC3T3-E1, indicating that the cells were still spreading and growing. While there were more cells on the surface 1%GO/CS/GP and 2%GO/CS/GP hydrogels that showed a spherical shape, indicating the cells were not completely attached to the substrate.

**Figure 9:**
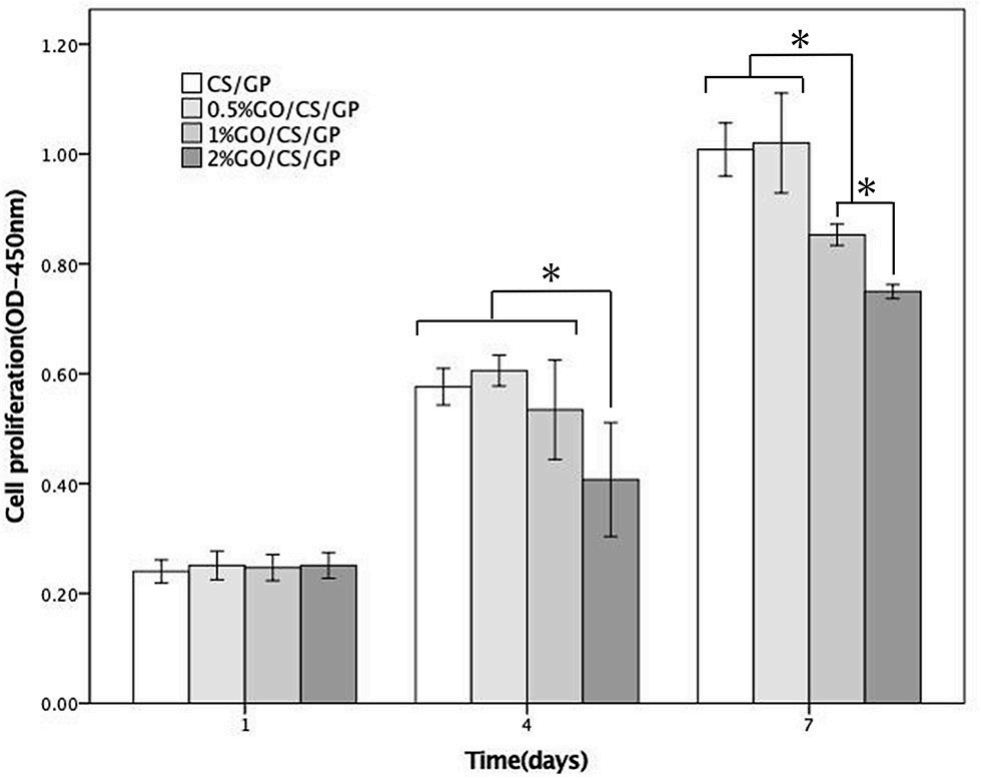
CCK-8 cellular proliferation assays of CS/GP 0.5%GO/CS/GP, 1%GO/CS/GP and 2%GO/CS/GP. *Statistically significant differences existing among the groups.

The results of CCK-8 assay of the samples at day 1, 4, and 7 were shown in Figure 9, revealing the proliferation of cells grown in all the hydrogels over the incubation period. However, there were differences among the OD values of groups at 4 d and 7 d, indicating the proliferation of cells was influenced by GO. At day 1, there was no significant difference of the OD values among the samples. At day 4 and 7, the OD values of 2%GO/CS/GP were significant lower than the other three samples. At day 7, the OD value of group 1%GO/CS/GP was lower than those of CS/GP and 0.5%GO/CS/GP, and the OD value of 2%GO/CS/GP was even lower.

**Figure 10:**
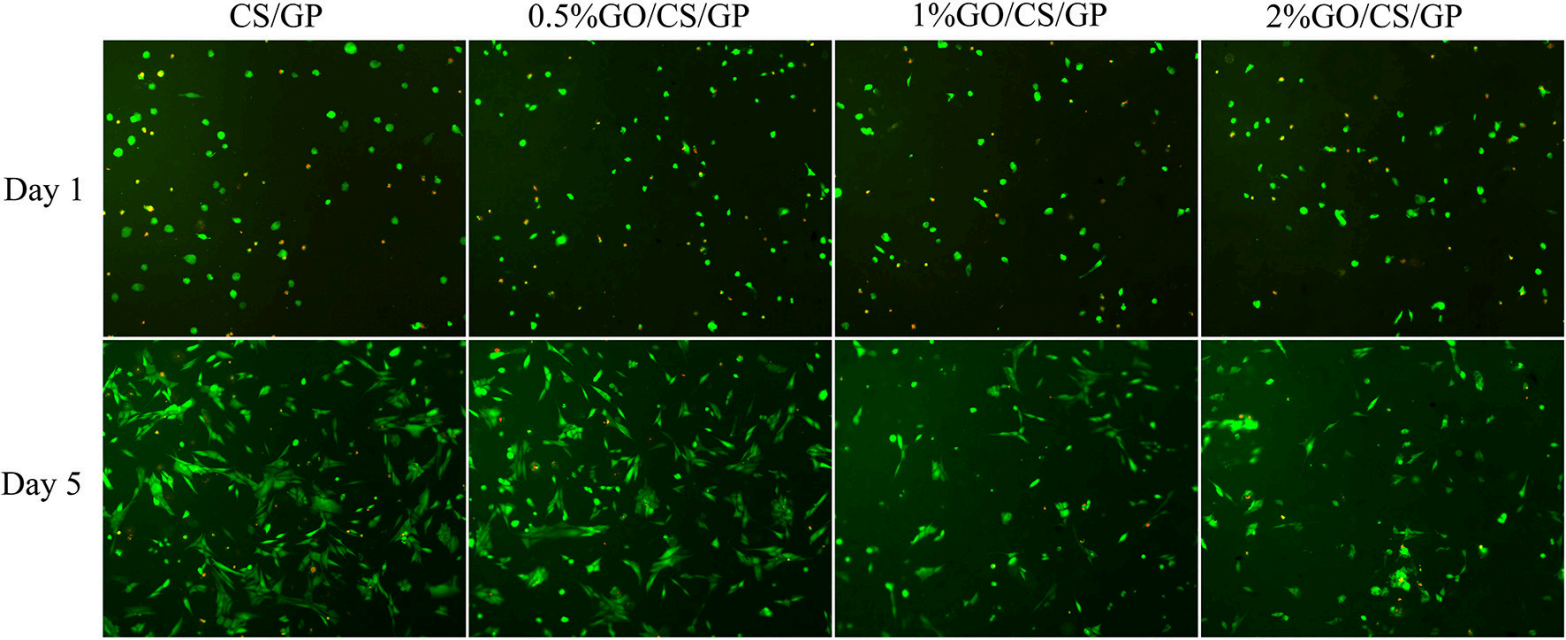
Fluorescence micrographs stained by Calcein-AM/PI illustrating the cellular viability on the hydrogels after incubation for 1 day and 5 days. Live cells were stained green and nucleus of dead cells were colored red.

Results of live/dead (Figure 10) staining revealed that most cells survived in all hydrogels, but there was no obvious difference in cell morphology and viability among the groups at d 1. After 5 days, it could be observed that cells in CS/GP and 0.5%GO/CS/GP spread better and showed a typically spindle-shaped morphology. There were less live cells in 1%GO/CS/GP and 2%GO/CS/GP and the proportion of spherical-shaped cells was higher. These images suggest that hydrogel containing small amount of GO (0.5%) showed no difference in cell attachment, viability and proliferation compared with CS/GP hydrogel.

## Discussion

In the present study, chitosan/β-Glycerophosphate hydrogels with different content of graphene oxide were fabricated through thermal-induced gelation at physiological pH and temperature, indicating that such materials can be possibly used as injectable *in situ* scaffold for tissue engineering.

### Mechanism of gel formation

**Figure 11:**
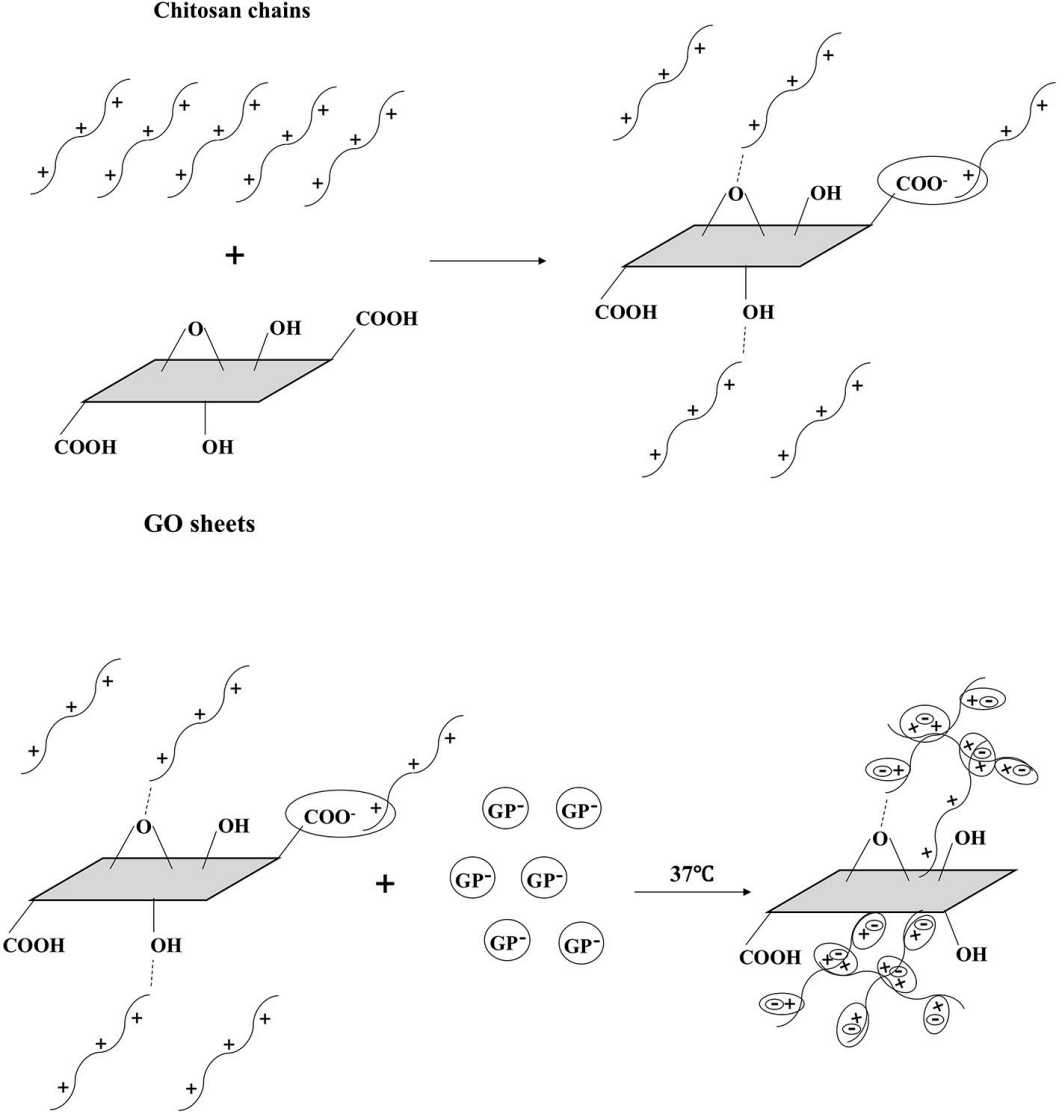
Schematic illustration of gel formation at 37°C. GO and CS were interacted by H-bonding and electrostatic bonding in the homogeneous codispersion. With the addition of GP, the repulsion of pronated CS was weakening due to the CS-GP electrostatic attractions via amino and the phosphate groups. When the temperature rises, the hydrophobic effect of CS chains becomes dominant. CS chains entangle with each other and the gel forms. GO sheets reinforce the hydrophobic effect, inducing the faster gelation.

GP is a key factor for gel formation. Previous studies reported that higher content of GP induced faster hydrogel formation or gelation at lower temperature (Cho et al., 2006). However, it has also been suggested that high concentrations of GP can be detrimental to cell viability and proliferation (Ahmadi and de Bruijn, 2008; Wang and Stegemann, 2010). The reason may be that high concentrations of GP avoidably dissolves and diffuses into the surrounding culture medium or tissue fluid, causing osmosis pressure change, which will lead to cell activity decrease or even cell death (Song et al., 2017). In order to initiate the gelling process at body temperature while avoiding causing cytotoxicity, the concentration of 6 wt% GP was chosen to prepare hydrogel in this work. The gelation mechanism of CS/GP system has been explained by many studies in literature. Briefly, there are three effective interactions involved in the sol/gel transition: (1) the electrostatic attractions between chitosan and β-glycerolphosphate via amino and the phosphate groups, respectively, (2) the chitosan interchain hydrogen bonding, and (3) the chitosan–chitosan hydrophobic interactions which could be enhanced by the structuring action of glycerol on water (Cho et al., 2006; Ahmadi and de Bruijn, 2008; Zhou et al., 2015). When adding GO, the molecular mechanism of gelation involves multiple interactions between CS, GP, GO and water (Figure 11). In addition to the three interactions mentioned above, in GO/CS/GP system, electrostatic attraction, hydrogen bonding and hydrophobic interaction also exist between GO and CS. When mixing GO dispersion and CS acetic acid solution, because of polycationic nature of CS in acid media and the negative charge on the surface of GO, electrostatic attraction can be achieved. Hydrogen bonding can be formed between the amino and hydroxyl groups in the unit of CS and the oxygen-containing groups on the GO. These two interactions between CS and GO could induce the truly homogeneous codispersion on the molecular scale (Yang et al., 2010). It has been confirmed that the hydrophobic effect is the main driving force for the heat-induced gelation of CS/GP system since the ratio of -NH${}_{3}^{+}$ in chitosan and -OPO(O^−^)^2^ in GP reduces and the hydrogen bonding are not predominant (Zhou et al., 2015). In the presence of GO, the sp^2^ bonded substrate could reinforce the hydrophobic effect of the system upon heating. As it shown in the rheological test and gelation time determination, hydrogel containing more GO had lower gelation temperature and faster gelation process.

### Properties of the hydrogels

The results of rheological tests revealed that the gelling process of all groups started around the body temperature and the results of gelation time indicated that all the hydrogels could be formed in several minutes at 37°C, which is of much importance in clinical application. When injecting the heat-induced *in situ* gelling system into irregular-shaped bone defect (e.g., oral and maxillofacial region), the material could well adjust to defect zone due to its fluidity. Appropriate gel formation speed gives the practitioner enough time to inject or shape the gel to an ideal form. And the practitioner does not have to waste too much time on waiting for the gelation before the next surgery step. The gelation time of the hydrogels (several minutes) in this study was approximately equal to or less than the time needed for putting bone substitute into the bone defect and laying a membrane above it, which is a routine procedure of guided bone regeneration (GBR) technique in oral implantation. From the perspective of operability, the thermal-sensitive hydrogels offer a more convenient and less time-consuming alternative procedure compared with traditional treatments.

The SEM images demonstrated highly porous structure of the hydrogels. The interconnecting and stable internal channel system provides space for embedded cells (Mirahmadi et al., 2013). Appropriate pore size, porosity and desirable water uptake ability are beneficial for cell adhesion, ingrowth and proliferation. The pore diameters and porosities in all the types of hydrogels in this study were found to be appropriate for bone tissue engineering applications (Amaral et al., 2009; Depan et al., 2011). An interesting phenomenon was that the average pore diameters and the porosities of the hydrogels were not in relation to the water uptake abilities. The former two were in positive correlation to the amount of GO, while the latter was not. Nevertheless, the results of water uptake proved satisfactory water-absorbing ability of the hydrogels.

Proper mechanical properties are required for scaffolds in order to maintain their structure for tissues ingrowth. Previous studies reported the elastic modulus of CS/GO composite film prepared by solution casting method was increased even by adding a small amount of GO compared with the pure CS film as control (Fan et al., 2010; Han et al., 2011). In this work, results of rheological measurement and unconfined compression test revealed that the mechanical properties of the hydrogels were improved by the addition of GO. The storage (G′)/loss (G″) moduli of the hydrogels with GO evaluated by rheology test were 1.12 to 1.69 times that of CS/GP at the gelling temperature. The elastic modulus of hydrogels containing different amounts of GO have been increased by 1.76 to 2.94 times compared with pure CS/GP hydrogel. GO has a large specific surface to volume ratio and a unique two-dimensional structure which probably imposes a higher degree of geometric constraint with regard to the mobility of polymer chains, influencing binding interactions between molecules and subsequently enhances elastic modulus (Han et al., 2011). The properties of GO/CS/GP (including 0.5, 1, 2%) were acceptable for tissue engineering applications.

### Biological assays

Besides physical and chemical properties, the cytocompatibility of biomaterials is of much importance for tissue engineering as scaffolds are expected to promote cellular adhesion, proliferation and differentiation. CS is a well-known biopolymer for its biocompatibility and biodegradability. Because of its ability to support cell growth and to integrate with surrounding ECM, chitosan has been widely studied in biomedical field. GP is an organic compound naturally found in the body and has been shown as an osteogenic supplement for culturing bone marrow stem cells (Zhou et al., 2015). However, it has also been reported that high concentrations of GP, in particular above 10wt% can be detrimental to cell proliferation (Zhu et al., 2010). In this study, the concentration of GP was relatively low (6 wt%), and all the hydrogels were immersed in complete cell culture medium for three times, half an hour for each time before seeding cells into them in order to remove the diffused GP, preventing possible toxicity caused by GP. Results of the biological assessment in this study revealed that the CS/GP promoted cellular adhesion and proliferation for MC3T3-E1. However, the cytocompatibility and toxicity of GO are controversial. Some investigators indicated that GO demonstrated biocompatibility in a number of studies aiming for biomedical applications while the other reported adverse biological responses and cytotoxicity (Gurunathan and Kim, 2016; Ou et al., 2016). In current study, results of CCK-8 assay showed that cells kept proliferating in the incubation with the hydrogels but there was a difference among the groups. Cells incubated in the hydrogel with low content of GO (0.5%GO/CS/GP) exhibited a similar proliferation rate with cells in the hydrogel without GO, while the hydrogels with higher contents of GO (1%GO/CS/GP and 2% GO/CS/GP) had an inhibiting effect on the cell attachment and proliferation. The live/dead staining images of samples also revealed that there were less live cells on the hydrogels with higher content GO (1%GO/CS/GP and 2% GO/CS/GP) after 5 days. The concentrations of GO in each hydrogel sample were 100 μg/mL (0.5%GO/CS/GP), 200 μg/mL (1%GO/CS/GP) and 400 μg/mL (0.5%GO/CS/GP), respectively. For cell incubation, the final concentrations of GO in each well of the culture plate were one third of those in hydrogels as the volume of culture medium been calculated. The results were consistent with studies concerning the dose-dependent toxicity of GO. A study reported that GO nanosheets at the concentration of 20 μg/mL had no toxicity in A549 within 2 h, but a higher concentration (85 μg/mL) reduced the cellular viability to 50 % within 24 h (Chang et al., 2011). Another study also demonstrated that GO had no obvious cytotoxicity at low concentrations for 96 h on human neuroblastoma SH-SY5Y cell line, but the viability of cells sharply decreased to 20 % after treatment with 100 mg/mL GO for 96 h of incubation (Lv et al., 2012). It is important to bear in mind that the toxicity of GO is influenced by many physicochemical properties such as lateral dimensions, surface area, surface chemistry, surface charge, layer number, purity, particulate state and shape. In this study, the dose-dependent adverse effects of GO on the cellular viability and proliferation cells may be attributed to the dispersed GO. As the physical interactions between GO and CS can be interrupted by water, different ions and molecules of the culture medium or released from cells, the GO sheets would disperse in the medium again. Given that the lateral diameter of GO used in this work was too large to be taken up by the cells, the dispersed GO may influence the growth of cells by cutting through cell membranes directly or binding with the proteins of the medium through π-π stacking, preventing them absorbed by the cells (Akhavan and Ghaderi, 2010; Zhou and Gao, 2014). For the GO entangled by CS, there would be less opportunity to expose its sharp margin and hexagonal lattice. Hence, hydrogels with higher content of GO that may release more dispersed GO had an adverse effect on cell viability. The cytocompatibility of 0.5%GO/CS/GP was acceptable for biomedical application.

## Conclusion

The thermal-sensitive chitosan/β-glycerophosphate hydrogels reinforced by graphene oxide were fabricated at physiological pH and temperature. The hydrogels possessed highly porous structure with good ability of water uptake, which favors living cells ingrowth and water convection. The presence of GO within the hydrogel stiffened the scaffolds because of its large surface to volume ratio and a unique two-dimensional structure. The biocompatibility was associated with the content of GO assessed by SEM, CCK-8 test and live/dead staining. The hydrogel with lower content of GO (0.5%GO/CS/GP) not only had an enhanced mechanical property but also favored for cell attachment, viability and proliferation, exhibiting potentiality for application in regenerative medicine such as being used as *in situ* gel-forming materials for tissue engineering.

## Author contributions

HQ carried out the overall experiment and drafted the manuscript. XP and JW participated in the design of the study and coordination. TW helped with the figures and data statics. QW and XP supervised this study. XP and XG revised the manuscript. All authors have read and approved the final manuscript.

### Conflict of interest statement

The authors declare that the research was conducted in the absence of any commercial or financial relationships that could be construed as a potential conflict of interest.
